# Obesity among Iranian Adolescent Girls: Location of Residence and Parental Obesity

**DOI:** 10.3329/jhpn.v28i1.4524

**Published:** 2010-02

**Authors:** Mohsen Maddah, Bahareh Nikooyeh

**Affiliations:** Department of Human Nutrition, School of Public Health, Guilan University of Medical Sciences and Health Services, Rasht, Iran

**Keywords:** Adolescents, Cross-sectional studies, Obesity, Overweight, Iran

## Abstract

This cross-sectional study was conducted to investigate the prevalence and predictors of overweight and obesity by location of residence among randomly-selected 2,577 urban school girls aged 12–17 years in Rasht, Iran. Data on age, frequency of skipping breakfast per week, physical activity, hours of television viewing, self-perception about body condition, and home address were collected. Birthweight of the girls, educational levels of parents, weights and heights of parents, and employment status of mothers were asked to the parents using a self-administrated questionnaire. The overall prevalence of overweight and obesity in this population was 18.6% and 5.9% respectively. Overweight or obesity was more common among girls from low-income areas compared to high-income areas (21.6% vs 17.1%, p<0.001). Maternal education was positively related to overweight/obesity of the girls. Results of logistic regression analysis showed that risk of overweight/obesity was higher in girls whose either parent was overweight or obese. Furthermore, living in low-income areas and skipping breakfast were independently related to overweight/obesity. These data suggest that overweight and obesity are a public-health concern among school girls, especially in low-income areas in Rasht. Knowing risk factors in population subgroups is important for planners in the country because it helps target interventions.

## INTRODUCTION

Overweight and obesity are rapidly increasing among children and adolescents in developing countries (1, 2). Adolescence is one of the critical periods for obesity and is related to morbidity and mortality in adulthood (3–5). Similar to obesity among adults, overweight and obesity among adolescents are more common in lower socioeconomic groups than in higher socioeconomic groups in developed countries (6, 7). Overweight and obesity among adolescents by socioeconomic levels have been less studied in developing countries.

Iran is a middle-income country, experiencing rapid epidemiological transition (8), and the high prevalence of hypertension, obesity, and type 2 diabetes has been documented in the population (9). Obesity is now the most prevalent nutritional disease among children and adolescents in Iran (10, 11). Results of recent studies showed that metabolic syndrome is highly prevalent among adolescent girls in Iran (12). No data on the prevalence of overweight and obesity among adolescent girls across socioeconomic levels in Iran are available. The knowledge on the prevalence of overweight and obesity and its determinants can help implement population-based preventive measures.

The main objective of this study was to provide current data on the prevalence of overweight and obesity among school girls by location of residence, socioeconomic status, and maternal educational levels in Rasht. We also aimed at exploring contribution of some lifestyle factors to these differences in this population.

## MATERIALS AND METHODS

The study was designed to evaluate the current status of overweight/obesity by location of residence and its correlates among school girls in urban areas of Rasht in northern Iran. The study population was school girls aged 12–17 years living in Rasht, the main city of Guilan province. During October 2006–March 2007, 2,610 school girls were randomly selected from low- and high-income areas in Rasht with no exclusion criteria. Rasht city was divided into five areas based on the type and price of house. One area known for its luxury houses where the rich live was considered a high-income area, and two areas where price of house was lower compared to other areas were considered low-income areas in this study. Twenty-five schools from low-income areas and 15 schools from high-income areas were randomly selected. Ten percent of students in each class were systematically selected using the class list. Selection of students was initially made by level of education (class), not by age. Since the age of 33 students was not in the range of the study protocol, they were excluded, and 2,577 observations were included in data analysis.

Data on age, frequency of skipping breakfast per week, physical activity, hours of television viewing or video game, birth-rank, self-reported body-weight, self-perception about body condition, and home address were collected using a self-administrated questionnaire. Information on birthweight of the girls, parental education levels, and employment status of mothers was gathered through a self-administrated questionnaire given to the parents. Original weight (defined as body-weight before getting married) and current body-weight and height of parents were self-reported. Frequency of breakfast was recorded by asking the question: how many times during a week do you take breakfast? Response categories were ‘seldom’ and ‘most times per week’. Self-perception about body condition was evaluated by asking “How do you think about your body-weight? The responses were categorized as ‘overweight’, ‘normal weight’, and ‘I have no idea’. A questionnaire on physical activity was also developed for the study that asked the participants to recall the number of hours per week if they had participated in any structured physical activity or team sport in the last six months. Response categories ranged from 0 to 8+ hours per week. The participants were asked about the time spent in commuting between home and school under five time-categories, such as less than five minutes per day, 5–15 minutes per day, 15–30 minutes per day, 30–45 minutes per day, and more than 45 minutes per day. Time spent in television watching and computer/video games was recorded for each day of a typical week and divided into three time-categories, such as <3 hours per day, 3–5 hours per day, and >5 hours per day. Anthropometric measurements of the girls were performed in light dress and without shoes in the morning. Body-weight was measured to the nearest 0.1 kg using a balanced-beam scale, and height was measured to the nearest 0.5 cm with the girls standing up and head, back, and buttock on the vertical land of the height-gauge. Self-reported body-weight of the girls was categorized as ‘I do not know’, ‘correct’ (if the reported weight was ±2 kg of the measured body-weight), and ‘incorrect’. Age and sex-specific body mass index (BMI) cut-offs, proposed by the International Obesity Task Force (IOTF), were used for defining overweight and obesity (13). Underweight was defined as BMI less than the 5^th^ percentile of the World Health Organization cut-offs. Parental overweight and obesity was defined as BMI equal or greater than 25 kg/m^2^ and 30 kg/m^2^ respectively.

### Statistical analysis

The differences in the prevalence of overweight and obesity were tested using chi-square statistics. In data analysis, level of education of mothers was classified as less than five years of schooling, 5–11 years of schooling, high school diploma (12 years schooling), and college study (>12 years of schooling). Employment of mothers was classified as housewife or employed. Logistic regression analysis was used for determining the predictors of overweight and obesity among the study girls.

Values were given as mean and 95% confidence intervals. The p values less than 0.05 were considered the level of significance. Analyses were performed using the SPSS software (version 10.01) for Windows (SPSS Inc® headquarter, Chicago, IL, USA).

### Ethics

All the parents gave written consent for participation in the study. The Ethical Committee of the Guilan University of Medical Sciences approved the study protocol.

## RESULTS

The mean values of BMI and the prevalence of overweight and obesity among the girls as a function of age, maternal education, and location of residence are presented in Table 1. Since there were more intermediary schools (grade 6 to 8) than high schools for girls (grade 9 to 11) in Rasht, more young girls than older girls were randomly selected. The overall prevalence of overweight and obesity was 18.6% and 5.9% respectively. The overall prevalence of underweight was 10.6% in this population. The prevalence of overweight/obesity was higher among girls with more-educated mothers than among girls with less-educated mothers (Table 1). In this study, overweight/obesity was more common in low-income areas than in high-income areas. The results also showed that those school girls who usually skipped breakfast were more at risk of overweight/obesity. 43.4% of the girls were not aware of their body-weight. Moreover, 29.1% had no idea in response to the question “How do you think about your body-weight?”, 16.4% of the girls considered themselves as overweight, and 87% of the girls did no exercise during the past week.

**Table 1.** Body mass index, overweight, and obesity in study adolescent girls, by their age, location of residence, and skipping breakfast

Variable	BMI (kg/m2) (95% CI)$	Overweight (%)	Obesity (%)
Age (years)			
12 (n=647)	19.1 (18.8–19.5)	19.8	5.0
13 (n=596)	20.3 (19.9–20.7)	20.5	7.6
14 (n=619)	20.5 (20.1–20.8)	16.0	5.1
15 n=331)	21.8 (21.3–22.3)	17.3	7.3
16 (n=201)	21.9 (21.3–22.6)	19.2	4.0
17 (n=183)	21.7 (20.9–22.5)	19.3	3.6
Location of residence			
High-income (n=1,520)		17.1*	8.0
Low-income (n=1,057)		21.6	8.5
Maternal educational levels (years)			
<5 (n=865)		16.9	4.3
5–11(n=732)		17.2	4.8
12 (n=822)		19.5	7.4
>12 (158)		25.3	7.0
Eating breakfast			
Regular (n=1,208)		17.1**	4.0
Not regular (n=1,369)		19.8	7.0

$Data for BMI are means with 95% confidence interval values given in parentheses;

*p<0.001 prevalence of overweight in low- and high-income areas was significantly different;

**p<0.001 prevalence of overweight was significantly higher in those who usually skipped breakfast than those who usually had their breakfast in the home;

BMI=Body mass index;

CI=Confidence interval

Overweight and obesity among mothers were 43.2% and 27.3% respectively. Overweight and obesity among fathers were 43.3% and 12.3% respectively. Specific weight gain after marriage was reported by 2,113 mothers and 1,984 fathers respectively. The mean weight gain by mothers and fathers was reportedly 15.3±10.4 and 10.4±9.4 kg over a 8–15-year period respectively. The figure shows that overweight and obesity were most common in those girls whose both the parents were overweight or obese.

**Fig.**
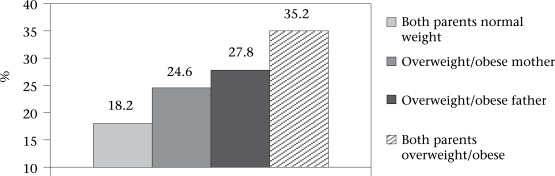
Prevalence of overweight/obesity among adolescent girls by weight status of their parents

The results of logistic regression analysis showed that risk of overweight/obesity among the school girls were independently related to parental overweight/obesity, location of residence, and skipping breakfast (Table 2).

**Table 2.** Logistic regression analysis of potential risk factors for overweight/obesity among study girls adjusted for each variable: maternal educational level, TV viewing (hour per day), birth-rank, employment of mothers (employed mothers), residency in low- or high-income areas, maternal overweight/obesity, paternal overweight/obesity, walking (hour per day), skipping breakfast as categorical variables, and age and birthweight as continuous variables

Variables	β±SE	OR (95% CI)	p value
Variables in model			
Maternal overweight	0.43±0.21	1.5 (1.08–2.30)	0.04
Maternal obesity	0.77±0.23	2.1 (1.31–3.42)	0.001
Paternal overweight	0.57±0.18	1.7 (1.22–2.51)	0.002
Paternal obesity	0.70±0.25	2.0 (1.25–3.36)	0.05
Low-income area	0.56±0.24	1.7 (1.09–2.81)	0.02
Skipping breakfast	0.35±0.16	1.4 (1.09–1.93)	0.002
Constant	0.73±0.1	_	0.001
Variables not in model			
Age of girls	−0.05±0.09	0.94 (0.79–1.12)	0.513
Employment of mothers	−0.28±0.30	0.75 (0.41–1.36)	0.351
Birthweight	0.03±0.42	0.65 (0.40–1.23)	0.49
Birth-rank	−0.01±0.04	0.33 (0.02–1.33)	0.29
Walking	−0.02±0.22	0.97 (0.41–1.36)	0.48
TV viewing	0.03±0.41	0.81 (0.34–1.61)	0.61

CI=Confidence interval;

OR=Odds ratio;

SE=Standard error;

TV=Television

## DISCUSSION

The prevalence of overweight and obesity is already high among Iranian women, and it ranged from 55% to 75% in different parts of Iran (15). Results of a recent study showed that Iranian women were not behind men regarding coronary artery disease (CAD) as they developed CAD at the same age (51 years) as Iranian men did (16). Will this generation of Iranian girls be more at risk of chronic diseases than their parents are? Many studies have documented the impact of adolescent obesity on adult morbidity (17, 18). The present study highlighted the importance of overweight and obesity among adolescent girls in Rasht city as a public-health issue.

Studies in developed countries have demonstrated a negative relationship between socioeconomic status and overweight among adolescents (6, 7). There is, however, little information on the association of socioeconomic status with overweight among adolescents in developing countries. Although location of residence may not be an accurate index of economic status, it is helpful when information on income cannot be collected. Many Iranian families have a second job, and income is usually under-reported by them**!** The present study has shown that the girls living in low-income areas were more prone to overweight or obesity than were the girls living in high-income areas. On the other hand, overweight and obesity were positively related to maternal education. Our findings showed that more-educated women were more likely to reside in low-income areas. In Iran, standard of living is low due to international sanctions and internal inflation, and many well-educated people have no employment opportunities (19). In multivariate analysis, location of residence was independently related to overweight after controlling for other variables, including educational level of the mother.

It has been previously reported that there is less social pressure for conforming to an ideal perception about body condition in Iran, an Islamic country (20). Iranian women and adolescent girls are less engaged in activities during leisure, and their Islamic dressing style might have made them less concerned regarding dieting and physical activity. The fact that a considerable proportion of the study girls was not aware of their body-weight confirms that what Iranian girls think of ideal body-weight is possibly different from their counterparts in Western societies. More detailed studies are needed to clarify whether they are less likely to perceive themselves as overweight than do their peers in Western countries.

The results of this study indicate that, when a broad range of factors were taken into consideration simultaneously, skipping breakfast, location of residence, and parental overweight and obesity had the largest association with overweight/obesity in these high school girls. Skipping breakfast has been reported to be associated with overweight/obesity in children (21, 22) but research on this subject remains inconclusive as a longitudinal study showed that skipping breakfast was not related to weight gain in adolescents (23). The findings of our study showed that skipping breakfast was positively related to overweight/obesity after controlling for other measured factors. We have recently shown that skipping breakfast is related to consumption of energy-dense, less-nutritious snacks during school hours (24).

Weight status of parents and especially maternal overweight have been reported to be related to overweight/obesity in adolescents in Western countries (25, 26). The results of the present study showed that overweight and obesity in either parents predicted overweight/obesity in adolescents. In addition to genetic resemblance, family members have similar behavioral risk factors associated with overweight and obesity (27). In this study, data on post-marriage weight gain were collected to show how parents and their children are getting fat in the same environment. These results suggest that the changes in lifestyle should be aimed at all members of a family.

There are no data on the physical activity patterns of Iranian adolescent girls. Results of a study showed that activities during leisure are extremely low among Iranian adult women due to religious reasons and lack of recreation facilities (20). The results of the present study showed that most of these girls did no exercise during the past week, and the average time spent on watching television was quite high (3.5±1.7 hours per day).

There are possible limitations to this study. Parental body-weight and height were self-reported in this study while there are no data to show whether self-reported weight and height is reliable in Iran. Besides, no direct data on economic status, i.e. income, were collected in this study.

In conclusion, chronic diseases, including CAD and diabetes, are the main causes of mortality in Iran (28). Available data showed that Iranian women and girls are much more at risk of obesity and related co-morbidities than are Iranian men and boys (9, 29). The hypothesis that social and religious factors, such as dressing style of Iranian girls in public and having less social pressure for conforming to an ideal weight, are related to the high prevalence of obesity in this community should be tested in future studies. Preventing obesity in children and adolescent girls should be regarded as an important public-health priority in Iran. Modifying the current ‘obesegenic’ environment, especially in low-income areas, is crucial for combating this epidemic. The results of the present study suggest that students should be encouraged to eat breakfast. Moreover, parental overweight and obesity as a predictor of overweight in the girls indicates that changes in the lifestyle need to be considered at the family level. Detailed research on this topic is strongly recommended.

## ACKNOWLEDGEMENTS

This work was financially supported by the Guilan University of Medical Sciences, Rasht, Iran. The authors thank the students and their parents for their participation in the study. The authors appreciate the school personnel for their cooperation in collecting the data.
